# Touch screen assays of behavioural flexibility and error characteristics in Eastern grey squirrels (*Sciurus carolinensis*)

**DOI:** 10.1007/s10071-017-1072-z

**Published:** 2017-01-27

**Authors:** Pizza Ka Yee Chow, Lisa A. Leaver, Ming Wang, Stephen E. G. Lea

**Affiliations:** 1 Centre for Research in Animal Behaviour, Psychology Department, University of Exeter, Exeter, EX4 4QG UK; 2 Division of Biostatistics and Bioinformatics, Department of Public Health Sciences, Penn State College of Medicine, Hershey, PA USA

**Keywords:** Reversal learning, Inhibition, Attention, Squirrels, Flexibility

## Abstract

Behavioural flexibility allows animals to adjust their behaviours according to changing environmental demands. Such flexibility is frequently assessed by the discrimination–reversal learning task. We examined grey squirrels’ behavioural flexibility, using a simultaneous colour discrimination–reversal learning task on a touch screen. Squirrels were trained to select their non-preferred colour in the discrimination phase, and their preferred colour was rewarded in a subsequent reversal phase. We used error rates to divide learning in each phase into three stages (perseveration, chance level and ‘learned’) and examined response inhibition and head-switching during each stage. We found consistent behavioural patterns were associated with each learning stage: in the perseveration stage, at the beginning of each training phase, squirrels showed comparable response latencies to correct and incorrect stimuli, along with a low level of head-switching. They quickly overcame perseveration, typically in one to three training blocks. In the chance-level stage, response latencies to both stimuli were low, but during initial discrimination squirrels showed more head-switches than in the previous stage. This suggests that squirrels were learning the current reward contingency by responding rapidly to a stimulus, but with increased attention to both stimuli. In the learned stage, response latencies to the correct stimulus and the number of head-switches were at their highest, whereas incorrect response latencies were at their lowest, and differed significantly from correct response latencies. These results suggest increased response inhibition and attention allowed the squirrels to minimise errors. They also suggest that errors in the ‘learned’ stage were related to impulsive emission of the pre-potent or previously learned responses.

**Electronic supplementary material:**

The online version of this article (doi:10.1007/s10071-017-1072-z) contains supplementary material, which is available to authorized users.

## Introduction

Behavioural flexibility is the ability to adjust behaviours according to environmental demands or changes. Such flexibility is important for survival both individually and for species as a whole. For example, individuals that show high flexibility in innovation obtain immediate benefits on fitness through obtaining a food source (Dukas 2013), or increased mating success (e.g. Cole et al. 2012, but also see Isden et al. 2013). High flexibility, as seen in the use of novel foraging techniques (Sol et al. 2013), is correlated with a higher number of species per parvorder (among birds: Nicolakakis et al. 2003), invasion success (Sol et al. 2002, 2008) and adaptation to city life (see review by Sol et al. 2013). Such fitness pay-offs predict that natural and sexual selection will favour behavioural flexibility and hence highlight the importance of understanding the mechanisms that support flexibility.

A discrimination–reversal learning task (Shettleworth 2010 p. 210–211) or simultaneous discrimination–reversal learning is commonly used to measure behavioural flexibility. This task has been applied in many animal models. Examples among vertebrates include, to name a few, in cynomolgus monkeys, *Macaca fascicularis* (Voytko et al. 1994), rhesus monkeys, *Macaca mulatta* (Bartus et al. 1979; Rapp 1990), rats (Bussey et al. 1997; Chudasama and Robbins 2003; Hu et al. 2006), pigeons, *Columba livia* (Bingman et al. 2008), kea, *Nestor notabilis* (O’Hara et al. 2015), zebra finches, *Taeniopygia guttate* (Brust et al. 2013), zebrafish, *Danio rerio* (Colwill et al. 2005), guppies, *Poecilia reticulata* (Lucon-Xiccato and Bisazza 2014), tropical arboreal lizards, *Anolis evermanni* (Leal and Powell 2012). Increasingly, there are also corresponding studies among invertebrates such as hawkmoths, *Macroglossum stellatarum* (Kelber 1996), bumblebees, *Bombus terrestris* (Raine and Chittka 2012) and jumping spiders, *Marpissa muscosa* (Liedtke and Schneider 2014). This simultaneous discrimination–reversal learning task involves two stimuli that take different values on some sensory modality (e.g. two different colours or shapes for vision, two distinct odours for olfactory or two locations for spatial navigation; see review by Izquierdo and Jentsch 2012) and requires animals to first associate one stimulus with reward and another with no reward. Once the animal reaches a stringent criterion, the reward contingency is reversed, so that the previously non-rewarded stimulus becomes rewarded and the previously rewarded stimulus becomes non-rewarded. Flexibility is measured as the number of errors or the number of trials taken to reach the learning criterion; individuals that are considered as higher flexibility make fewer errors or take fewer trials to reach the criterion than those individuals that make more errors or take more number of trials to reach the criterion (Brady and Floresco 2015). Success on this task requires a series of adjustments when the reward contingency changes: individuals have to notice the change, inhibit their previously learned response, overcome the learned but now irrelevant association with the non-rewarded stimulus and pay attention to the new association (Boulougouris et al. 2008). Such adjustments involve learning mechanisms such as attention and response inhibition (see review by Nilsson et al. 2015).

Assessing these mechanisms has proven problematic, however, because different studies have used the same measurements for attention or response inhibition as have been used to measure flexibility. For example, the number of errors and number of trials taken to reach the learning criterion have been used to measure both inhibitory control or inhibition (e.g. Tapp et al. 2003; see review by Izquierdo et al. 2016) and attention (e.g. Birrell and Brown 2000). The fact that the same measures have been used for these two distinct concepts reflects the close relationship between learning mechanisms and flexibility. It makes it difficult, however, to elucidate reasons for success or failure in the reversal task. For example, it is not clear whether poor performance is due to low inhibitory control or lack of attention, unless studies incorporate invasive methods (see reviews by Boulougouris et al. 2008; Clark et al. 2004; Clarke et al. 2004; Tait and Brown 2007).

Rather than using the broad outcome of performance to understand learning mechanisms, a detailed analysis of the observed behavioural responses may provide useful information pertaining to the underlying psychological state or cognitive processes of individuals during the learning process. Indeed, behavioural responses such as head-switching, a behavioural response in which animals turning their heads back and forth at a two-choice point (e.g. Gellermann 1933; Griesbach et al. 1998; Hu and Amsel 1995; Hu et al. 2006; Muenzinger 1938; Redish 2016; Tolman 1938; Kemble and Beckman 1970) and response latencies to a stimulus (e.g. Alsiö et al. 2015; Arnall et al. 2010; Bryce and Howland 2015; Clarke et al. 2004) have been used to infer the psychological state of individuals in the discrimination learning task. For example, Tolman (1938) noted that at the initial stage of the discrimination phase, rats increased the rate of head-switching in front of a Y-maze during a spatial discrimination task. This could be interpreted as ‘confusion’ or ‘hesitation’ in making a choice. However, Gellermann (1933) observed that chimpanzees and children increased head-switching near the end of a form discrimination task, accompanying an increased number of correct responses. Hu et al. (2006) also showed similar results in rats that were learning a visual discrimination task using a Y-maze, and Hu and Amsel (1995) showed that a lower rate of head-switching is related to slow learning progress. Gellermann (1933) suggested that the change in head-switching that he noted is related to attention to the characteristics of the relevant stimulus on a task. Another behavioural response, increased response latency towards an incorrect stimulus, as shown by male marmosets, *Callithrix jacchus,* has been suggested to be related to low motivation, distraction or uncertainty (LaClair and Lacreuse 2016), while a decrease in total response latency to a stimulus, as shown when mice make more correct responses in the reversal learning task (Arnall et al. 2010), has been held to reflect individuals’ learning of the new reward contingency. There is also evidence suggesting that behavioural responses may vary between different stages of learning, for example, between early and late stages of learning (e.g. Bryce and Howland 2015; see review by Nilsson et al. 2015; Izquierdo et al. 2016) and/or between the perseveration, chance-level and ‘learned’ stages identified by Jones and Mishkin (1972) and used in recent study such as LaClair and Lacreuse (2016). The analysis of errors by learning stage could allow investigators to disentangle perseveration (i.e. an inability to overcome a previously learned reward contingency) from other factors such as an inability to form new associations despite the changed reward contingency (e.g. LaClair and Lacreuse 2016; see review by Nilsson et al. 2015; Izquierdo et al. 2016).

In this study, our primary interest was to examine the behavioural flexibility of grey squirrels (*Sciurus carolinensis*) in a colour discrimination–reversal learning task on a touch screen. We examined squirrels’ flexibility by recording the number of errors in three learning stages (perseveration, chance level or ‘learned’) for each training phase (discrimination and reversal phase). We also examined the characteristics of behavioural responses in each stage. Behavioural responses of particular interest were head-switching, which may reflect attentional shift or ‘confusion/hesitation’, and the choice response latencies, which may reflect motor response inhibition. In this reversal task, we used green and red as the colour cues because, although grey squirrels’ colour vision is dichromatic (Silver 1976; Carvalho et al. 2006), they have been shown to discriminate these colours in a field situation (Macdonald 1997). We initially determined squirrels’ colour preferences, or trained them to prefer a colour. Then, we trained them to overcome this preference in the discrimination phase, and we reversed the colour contingencies in the reversal phase. Hence, in this paradigm, response inhibition is expected to play a key role in both training phases. Based on a previous study that showed squirrels were capable of completing a serial spatial reversal task (Chow et al. 2015), we predicted that squirrels would complete this colour reversal learning task. That is to say, the number of errors should decrease with increased training blocks. However, we had no basis for predicting how attention or response inhibition would change in the course of learning, as this is largely unexplored in squirrels. We can outline some possible patterns of behaviour, and their implications, as follows:

1. If the primary difficulty is in overcoming an unconditional preference or a previously trained association with reward, the perseveration stage should be longer (involving more blocks and more errors) than later stages of learning.
2. LaClair and Lacreuse (2016) argued that, if the effect of non-reward to a previously preferred or rewarded stimulus is to leave the subjects confused, we would expect increasing response inhibition, and hence increasing latencies to the incorrect stimulus, as learning progresses. Presumably the same should be true of the latency to the correct stimulus. That is to say, the response latencies to incorrect and correct stimulus should be comparable to each other if subjects are confused.
3. There are two, contradictory, bases for prediction of the trends in head-switching. Following Tolman (1938), we could predict that head-switching should be high in the initial stages of learning a visual discrimination task, which reflects ‘confusion or hesitation’, and then decrease as the learned stage is reached owing to diminishing confusion as the appropriate response is learned. Alternatively, following Gellermann (1933) and Hu et al. (2006), if performance depends on subjects actively comparing or learning the characteristics of the stimuli, we could predict low head-switching in the initial stages and then an increase across training blocks (Gellermann 1933; Hu et al. 2006), accompanying an increase in the proportion of correct choices (or decreased number of errors). We would also observe decreased response latencies when the learned stage is reached (Arnall et al. 2010).

We chose grey squirrels as a study species because we have previously examined squirrels’ flexibility both in problem solving (Chow et al. 2016) and in spatial cue use, a skill that has special adaptive value for them in caching, and revealed that they have no difficulty in completing a reversal learning task (Chow et al. 2015). As it has been shown by MacLean et al. (2014) that inhibitory control is shown in a range of species, we assumed that these mechanisms also exist in squirrels, although direct evidence of these mechanisms in squirrels comes mainly from their caching behaviours. Grey squirrels show inhibitory control by stopping digging and increasing the latency to start caching when conspecifics are present (Hopewell and Leaver 2008), and they are attentive to the presence of conspecifics (Hopewell et al. 2008) and heterospecifics (Schmidt and Ostfeld 2008) for the purpose of decreasing pilferage rate during caching.

## Methods

### Subjects and housing

Five captive grey squirrels, two females and three males with a mean age of 4 years old, housed at the University of Exeter participated in this study; see Table S1 for detailed biological information about each squirrel. Prior to this experiment, all squirrels had participated in caching studies (see doctoral thesis by Jayne 2014; Chow et al. unpublished data), but Squirrels 1 and 4 had experience in using the touch screen. On welfare grounds, squirrels were not food deprived during the experiment, and water was provided ad libitum. We ensured squirrels were motivated for the task by testing at each individual’s active foraging time and using rewards that were different from their daily diet (see Touch screen set up). Each day, motivation was further confirmed when the squirrels voluntarily went into the test room through an overhead tunnel that connected their home cage with the test room (see Hopewell et al. 2010 for detailed information about housing and test room arrangements). In the present study, squirrels’ overall participation rate was 100% with 90% completed blocks. Data collection for this study was conducted in two time periods, from November 2012 to January 2013 and from May to June 2013. This study was approved by the Ethical Review Group at the University of Exeter (no. 2012/533). Squirrels were treated in accordance with Association for the Study of Animal Behaviour guidelines on animal welfare and UK law.

### Touch screen set up

**Fig. 1:**
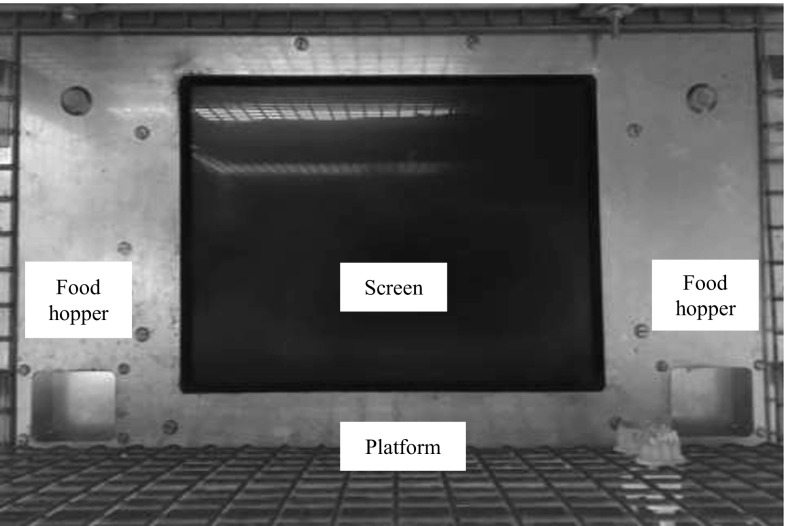
Touch screen set up for squirrels. The screen is at the centre with two food hoppers, one on each side. Stimuli are presented at the eye level of squirrels and correct stimulus leads to food delivery on the corresponding side

Figure 1 shows the touch screen panel that was used for this experiment. It was mounted on one wall of the test room, with its base approximately 2 m above from the floor. It included a 15-inch touch screen (Elo TouchSystems, Inc. Model: ET1546L-8UWA-1) and two recesses (Length: 6 cm × Width: 5 cm), one located on the left and one on the right side of the screen. Rewards of hemp seed, cashew nuts or pieces of breakfast cereal could be delivered to the recesses by motor-operated feeders. A wire mesh platform (52 cm × 28.5 cm) was attached just below the screen. Events on the screen were controlled by a computer located in a neighbouring area, using the Whisker control system (Cardinal and Aitken 2010) and a client program written in Visual Basic 6.

### Procedures

#### Pre-training

All five squirrels went through pre-training that was similar to that used by Wills et al. (2009) with pigeons; the pre-training was divided into four key stages, habituation, left or right side stimulus training, central stimulus training and hexagon training. In the habituation stage, intermittent food was delivered from both feeders unconditionally, allowing the squirrels to become habituated to obtaining food from them. This stage lasted for one day with 30 min. In the left or right side stimulus training, we used a side stimulus consisting of a white circle with 4.5 cm diameter, centred and 4.25 cm from either left or the right side of the screen. Sessions consisted of 60 trials (30 min/day for two days); the side stimulus was presented an equal number of times on the left and the right side, in a pseudo-randomised sequence. When a side stimulus was presented on the screen, naïve individuals received hand-shaping, being rewarded with a feedback beep and food when they went close to the screen. Experienced squirrels were required to nose-poke the stimulus once to activate the feedback beep. In both cases, as the beep sounded, the stimulus disappeared from the screen and the food dispenser delivered food immediately. Once squirrels had learned to poke the side stimuli, they then went through the central stimulus training stage (60 trials/day). In this stage, a central stimulus that was exactly the same as the side stimulus was presented at the eye level of squirrels. Squirrels were required to nose-poke the central stimulus to activate either side stimulus, and food was then delivered. This stage lasted for 30 min per day for two days. The final pre-training stage was hexagon training, aimed to increase the time that squirrels would remain engaged with the training. In this stage, poking the central stimulus exposed an array of twelve hexagons formed in a square shape with four hexagons on each of the four sides of the array. A poke at each hexagon led to that hexagon disappearing from the screen and the display of the nearest side stimulus, and hence to a reward; once all 12 hexagons had been removed, the central stimulus for the next trial was presented again. Squirrels completed five arrays within an hour each day for three days.

#### Pre-existing colour bias test

As we had no basis for assuming a pre-existing colour bias in squirrels, we gave the squirrels five trials of a ‘colour preference’ test before training. This test consisted of a pair of triangles (width × height: 3 cm × 3.2 cm), one pure red (RGB: 255, 0, 0) and the other pure green (RGB: 0, 255, 0). The colour pairs were presented on the touch screen at the eye level of the squirrels, 9 cm apart. The presentation of the colour pair was pseudo-random with one colour presented no more than three consecutive times on one side of the screen. Both colours were equally rewarded (one hemp seed); squirrels had to respond to both colours, to minimise any colour-reward associations acquired prior to the training. Colour bias was defined here as the colour that a squirrel chose first for three or more consecutive trials. Four squirrels (Squirrels 1, 2, 4 and 5) showed a bias towards green colour. Squirrel 3 showed no bias for either colour, although she made 3/5 non-consecutive choices towards green. In this case, we used one pre-training block with 60 trials to reinforce her colour preference to green. She showed 42/60 (70%) choices on green before going on to the training phase (two-tailed binominal exact test: *P* = 0.003). Accordingly, all squirrels went to the discrimination phase with a preference for green.

#### Training phase

Training involved two phases, a discrimination acquisition phase and a reversal phase. Squirrels received a block of 60 trials, lasting approximately 1 h daily, depending on the squirrel’s performance. Squirrels started each trial by nose-pressing a central stimulus before the same pair of stimuli as in the colour preference test were presented simultaneously. To avoid side biases, each colour was presented on each side of the screen 30 times and never more than three times consecutively on the same side. Response to the correct colour in each trial led to immediate food delivery (a hemp seed and a honey Cheerios^®^ or ¼ cashew) in the corresponding side recess. An incorrect response led to a 2-s time out during which responses had no scheduled consequences; the squirrels were then allowed to respond to the correct colour (correction trials). In the acquisition phase, we reinforced responses to the squirrels’ non-preferred colour (i.e. red +, green −). Training continued until a squirrel reached the learning criterion, 45/60 or more trials correct (75%) for two consecutive blocks (binominal exact test: *P* < 0.001). We then switched the reward contingency (i.e. red −, green +). Squirrels were then trained under the new reward contingency until they reached the learning criterion. Squirrel 4 did not reach the criterion after a month of training in the discrimination phase, but his performance reliably reached 70% or above. We adjusted his learning criterion to 70% (42/60 correct trials) for two consecutive blocks (two-tailed binominal exact test: *P* = 0.003), and this criterion was also applied for his reversal phase. Training ended each day when squirrels either completed the 60-trial block or did not respond for 20 min. All reaction times were recorded by the Whisker system (Cardinal and Aitken 2010). A camera was set adjacent to the touch screen platform and was connected to a camera control (ViewCommander 6) to live stream the performance on a computer screen. These behavioural responses were then recorded by a video camera that was set 60 cm away from the computer screen.

### Measurements

#### Flexibility

Flexibility was defined as the number of errors that squirrels made in each training phase. In each phase, we also used the error rate to divide the training blocks into three learning stages (perseveration, chance level and ‘learned’, see Jones and Mishkin 1972; LaClair and Lacreuse 2016; Izquierdo and Jentsch 2012). The perseveration stage included blocks (60 trials/block) in which squirrels made 39–60 errors, indicating retention of the previous reward contingency or the unconditional pre-potent responses. The chance-level stage included blocks with 22–38 errors, so there was no significant tendency to respond to either stimulus. The ‘learned’ stage consisted of blocks with 1–21 errors, including the blocks in which the learning criterion was met. The cut-off points of 21 and 38 were chosen to correspond to the 0.05 significance level for chance within a single block.

#### Behavioural responses

We measured three types of behavioural response and recorded them separately for each learning stage. The first behavioural response was head-switching, which was recorded whenever a squirrel turned its head between the two stimuli before making a choice, regardless of whether the choice was correct or incorrect. For example, a squirrel that switched its head from red to green to red colour showed two head-switches. The experimenter (the first author) analysed all head-switches on a frame-by-frame basis using Premiere Pro CS 6. Typically, the experimenter started recording a head-switch when a squirrel was facing towards one stimulus and the head movement in the next consecutive frames was moving towards the other stimulus (the degree of head movement ranged from 10° to 140°, depending on where was the squirrel sitting on the platform) and ended when the head movement stopped for at least three consecutive frames. We obtained the total number of head-switches for each block (60 trials) and the median of head-switching across blocks for each learning stage. The remaining two behavioural responses were the latency of first responding to the incorrect stimulus and the latency of first responding to the correct stimulus in each training phase. These response latencies were obtained from the Whisker system (Cardinal and Aitken 2010). For both latency measures, we obtained the median of latencies for each individual in each block and the median across blocks for each learning stage.

### Data analysis

We analysed data from completed blocks by the generalised linear mixed models (GLMM). We examined the effects of two factors, training phase (discrimination and reversal training) and learning stage (perseveration, chance level or learned), on the number of head-switches, the response latency to the incorrect stimulus and the response latency to the correct stimulus. Because the number of errors defined the learning stage, the analysis of errors only included the independent variable training phase. The distributions of response latencies to both correct and incorrect stimuli, and that of the numbers of errors deviated significantly from normality (Shapiro–Wilk tests, *P* < 0.001), and the number of head-switches showed a similar though non-significant tendency; accordingly, we followed the recommendations of Winer (1971, pp. 399–400) and log-transformed the latency measures and square root transformed the count measures. The Gaussian distribution could therefore be applied for all response variables. We included squirrels’ identity and training trials within blocks as random effects. Data analyses were conducted using package ‘lme4’ (Bates et al. 2015) and ‘glmm’ (Knudson 2015) in R (version 3.1.3). Results of all tests are reported as two-tailed with significance level set at *α* < 0.05. To prevent multiple pairwise comparisons inflating the Type I error rate, we used Bonferroni corrections to adjust the *P* values for the tests of pairwise comparisons between learning stages. These results are reported as two-tailed with significance level set at *α* <=0.025.

## Results

### Pre-existing colour bias in the discrimination phase

The initial colour bias towards green was confirmed by the first choice that squirrels made in the first 10 trials of the first block of the discrimination phase; all squirrels made eight or more choices of green.

### Performance in each training phase

**Fig. 2:**
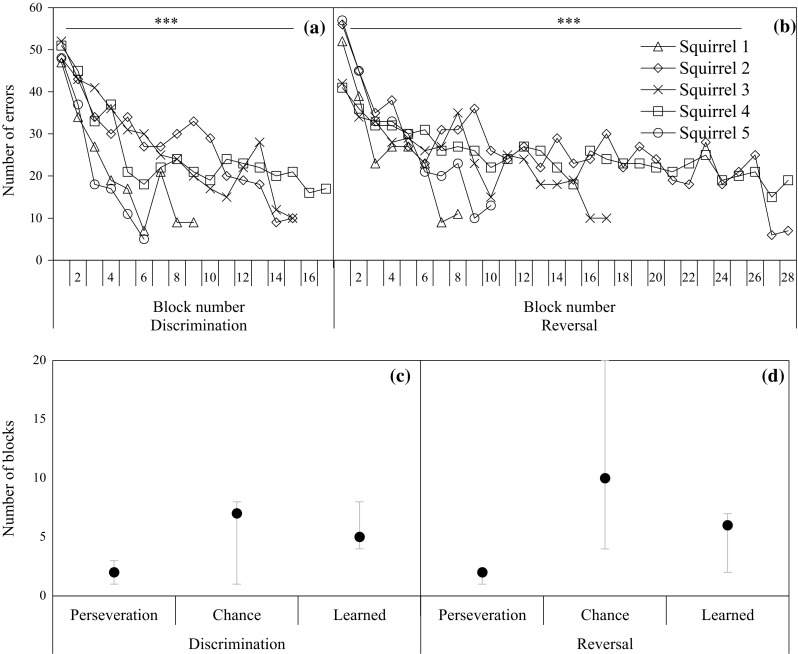
Number of errors squirrels made across blocks in **a** the discrimination phase and **b** the reversal phase. The median, minimum and maximum number of blocks that squirrels taken for each learning stages (perseveration, chance level, ‘learned’) in **c** the discrimination phase and **d** the reversal phase. *** < 0.001

Figure 2a, b shows the number of errors that squirrels made in both training phases. Squirrels decreased the number of errors (responses to the initially preferred colour) across blocks in the discrimination phase (*t*(57.5) = −9.41, *P* < 0.001) and the number of errors (responses to the previously rewarded colour) in the reversal phase (*t*(88.7) = −0.09, *P* < 0.001). The total number of errors that squirrels made across blocks was significantly higher (*t*(124.9) = 2.19, *P* = 0.030) in the reversal phase (Median = 17 blocks, 412 trials) than in the discrimination phase (Median = 15 blocks, 384 trials). Figure 2c shows the number of blocks that squirrels took in each learning stage in the discrimination phase. Squirrels used a median of two blocks at the perseveration stage, seven training blocks at the chance-level stage and five blocks for the learned stage (including the two blocks in which they reached the criterion). Figure 2d shows the number of blocks taken at each learning stage in the reversal phase. Medians for blocks were two for the perseveration stage, ten for the chance-level stage and six for the learned stage (including the two blocks in which the squirrels reached the learning criterion).

### Behavioural responses, training phase and errors

#### Response latency to the correct stimulus

**Fig. 3:**
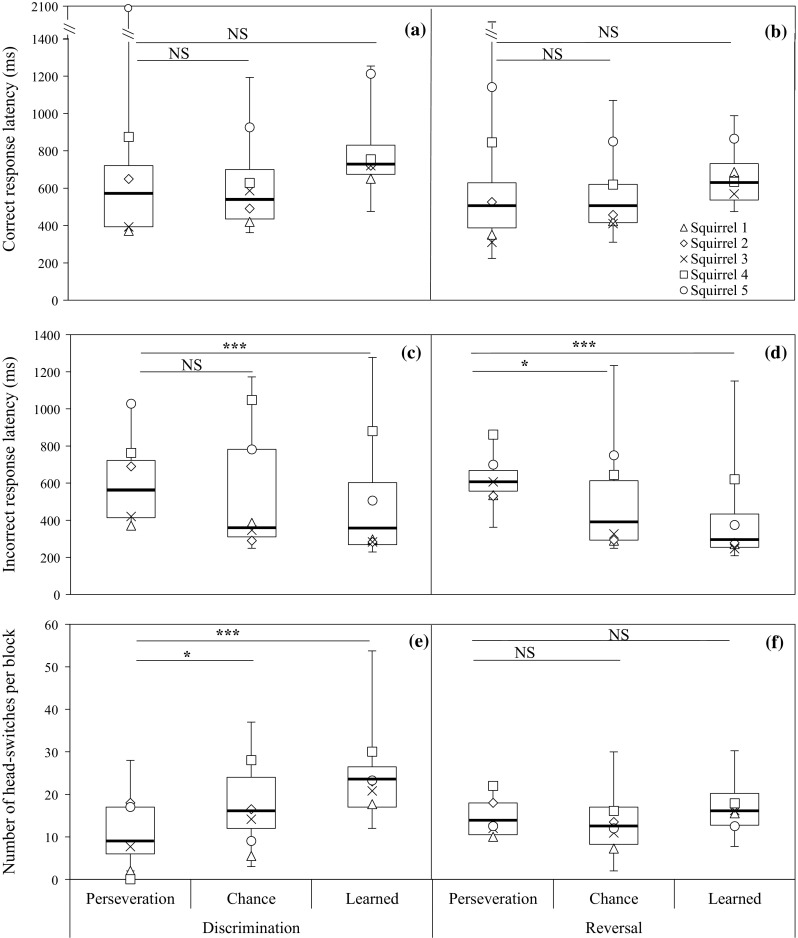
*Box plots* of each behavioural response for each training phase, broken down by three learning stages (perseveration, chance level and ‘learned’). The response latency of first choosing the correct stimulus (ms) in **a** the discrimination phase and **b** the reversal phase. The response latency of first responding to incorrect stimulus (ms) in **c** the discrimination phase and **d** the reversal phase. The number of head-switches per block (60 trials) in **e** the discrimination phase and **f** the reversal phase. * <0.05, *** <0.001

Squirrels did not show significant variation in correct response latencies across training blocks in the discrimination phase (*t*(57.0) = 1.40, *P* = 0.17), but significant increased response latencies across blocks were obtained in the reversal phase (*t*(86.9) = 2.89, *P* = 0.005). The response latency to the correct stimulus was lower in the reversal phase than in the discrimination phase, and this difference was significant (*t*(147.0) = −5.17, *P* < 0.001). The mean of median correct response latencies was 706 ms in the discrimination phase and 577 ms in the reversal phase. Figure 3a shows the response latency to the correct stimulus broken down by learning stages in the discrimination phase. Response latency to the correct stimulus was not significantly different between the perseveration and chance-level stages (*t*(54.9) = −0.86, *P* = 0.39) or between perseveration and learned stages (*t*(54.9) = 1.50, *P* = 0.14). Figure 3b shows the response latency to the correct stimulus for the reversal phase. Results showed no significant differences between perseveration and chance-level stages (*t*(84.5) = −0.44, *P* = 0.66), or between perseveration and learned stages (*t*(84.3) = 1.99, *P* = 0.050, Bonferroni corrected *P* > 0.025, NS). These results revealed squirrels showed comparable response latencies to the correct stimulus across the learning stages within each training phase.

#### Response latency to the incorrect stimulus

Incorrect response latencies significantly decreased across training blocks in the discrimination phase (*t*(56.7) = −4.91, *P* < 0.001) and in the reversal phase (*t*(86.6) = −4.17, *P* < 0.001). The reversal phase showed a significantly lower incorrect choice response latency than the discrimination phase (*t*(132.6) = −2.39, *P* = 0.018). The mean of median response latencies to the incorrect stimulus across individuals in the discrimination phase was 526 ms and in the reversal phase was 450 ms. Figure 3c shows the response latencies to the incorrect stimulus in the discrimination phase, broken down by learning stages. In this phase, chance-level error latencies and learned stage error latencies were lower than the perseveration stage error latencies. However, response latencies to the incorrect stimulus were not significantly different between the perseveration and chance-level stages (*t*(55.1) = −2.20, *P* = 0.031; adjusted *P* > 0.025, NS), but they did differ between the perseveration and learned stages (*t*(55.1) = −3.78, *P* < 0.001). Figure 3d shows the response latencies to the incorrect stimulus in the reversal phase, broken down by learning stages. As in the discrimination phase, response latencies to the incorrect stimulus were lower in the chance-level and learned stages than in the perseveration stage. Response latencies to the incorrect stimulus were significantly different between perseveration and chance-level stage latencies (*t*(32.6) = −3.43, *P* = 0.002) and between the perseveration and learned stage (*t*(34.1) = −5.08, *P* < 0.001). Overall, these results reveal that the squirrels decreased their response latencies to incorrect stimulus across the learning stages in each training phase, with the highest response latencies shown in the perseveration stage and the lowest in the learned stage.

In general, as Fig. 3a, c shows, the response latency was lower to the incorrect stimulus than to the correct stimulus in all three learning stages in the discrimination phase. The correct and incorrect response latencies were not significantly different in the perseveration stage (*t*(9.9) = −0.94, *P* = 0.37), though they were different during the chance-level stage (*t*(43.9) = −2.49, *P* = 0.017), and in the learned stage (*t*(50.0) = −7.04, *P* < 0.001). For the reversal phase, the response latency was lower to the incorrect stimulus than to the correct stimulus. As Fig. 3b, d show, as learning stages progressed, the difference between the latencies became larger. Correspondingly, no significant difference was obtained for the perseveration stage (*t*(10.0) = 0.63, *P* = 0.54), but significant difference was obtained for the chance-level stage (*t*(94.4) = −4.41, *P* < 0.001) and for the learned stage (*t*(33.9) = −8.55, *P* < 0.001).

#### Head-switching

Head-switching increased across training blocks both in the discrimination phase (*t*(59.2) = 3.42, *P* = 0.001) and in the reversal phase (*t*(70.0) = 2.28, *P* = 0.025). However, lower head-switching per block was observed in the reversal learning phase (mean of medians = 14) than in the discrimination phase (mean of medians = 20) and this difference was significant (*t*(146.6) = −4.64, *P* < 0.001). We further examined head-switching rate between the correct and incorrect stimulus in each training phase. To do so, we divided the number of head-switching that a squirrel exhibited during a trial by the response latency of the correct/incorrect stimulus of that trial. Head-switching rate was lower for the incorrect stimulus than the correct stimulus both in the discrimination phase (*t*(106.3) = −3.25, *P* = 0.002) and in the reversal phase (*t*(171.5) = −2.44, *P* = 0.016). This shows that low head-switching is related to errors. Figure 3e shows head-switches during the three learning stages in the discrimination phase. The perseveration stage, which included the blocks with the highest number of errors (and hence, lowest number of correct choices) showed significant difference in head-switches per block to the chance-level stage (*t*(55.3) = 2.40, *P* = 0.020) and the learned stage (*t*(55.4) = 5.07, *P* < 0.001). Figure 3f shows the rate of head-switching in the reversal phase. There were no significant differences between the perseveration stage and the chance-level stage (*t*(85.4) = −1.35, *P* = 0.18) as well as between the perseveration stage and the learned stage (*t*(84.4) = 0.56, *P* = 0.58).

## Discussion

In the present study, we examined grey squirrels’ behavioural flexibility using a colour discrimination–reversal learning task on a touch screen. Our results revealed that squirrels are flexible, in that they first overcame their colour bias and then overcame the previously learned reward contingency. We also provided evidence for how squirrels progressively decreased the errors they made in the task by analysing the behavioural assays of response inhibition (response latency to the incorrect or correct colour) and attention (number of head-switches) under each learning stage (perseveration, chance level and ‘learned’).

Not all animals can successfully overcome their colour preference in a simultaneous visual reversal learning task. For example, Leal and Powell (2012) showed that tropical arboreal lizards could not overcome their preference for black over white. Failure to complete a reversal learning task could be due to colour bias, but a frequent cause is low inhibition or inhibitory control. The design of this colour reversal learning task required squirrels to show inhibition in both training phases. At the start of each training phase, the squirrels showed a strong bias for their unconditional pre-potent colour (green) in the discrimination phase and to the learned rewarded colour (red) in the reversal phase. The fact that squirrels could overcome this bias and learned reward contingency was largely influenced by the squirrels’ capacity to show inhibitory control towards their preferred colour when it was not rewarded in the discrimination phase (Fig. 2a) and when their learned reward colour (initial non-preferred colour) was no longer rewarded in the reversal phase (Fig. 2b). This explanation is supported by our finding that response latency to the correct stimulus did not vary significantly in the discrimination phase and it increased across training blocks in the reversal phase, while the response latency to the incorrect stimulus decreased across blocks in both training phases.

At first glance, the fact that squirrels made more errors in the reversal phase than in the discrimination phase (Fig. 2) may suggest that errors could be due to inability to overcome the learned reward contingency (perseveration) or to learn the new reward contingency (Tait and Brown 2007, 2008). However, the fact that squirrels progressed from perseveration to chance-level stage in one to three training blocks in the discrimination phase (Fig. 2a) and in one or two blocks in the reversal phase (Fig. 2b) suggests that they were able to overcome the learned reward contingency; the squirrels quickly ‘noticed’ the lack of reward to the previously preferred or rewarded stimuli in both training phases. The fact that squirrels took most blocks in the chance-level stage (Fig. 2c, d) suggests that squirrels required substantial experience to form a new association corresponding to the current reward contingency. The analysis of behavioural responses further shows that making more errors is associated with low response inhibition to the incorrect stimulus and low head-switching. Between phases, we found that response inhibition, as measured by the latency of first choice to the incorrect stimulus, and head-switching, recorded as head turning back and forth between stimuli, were lower in the reversal phase than in the discrimination phase. The increased errors made in the reversal task are unsurprising, given the design of the simultaneous reversal learning task which presents the newly rewarded stimulus alongside the previously rewarded stimulus. It follows that the previously rewarded stimulus may become a distraction for individuals. These two behavioural responses may reflect the mechanisms of attention and response inhibition, with low levels of both associated with increased errors. But were squirrels ‘hesitating’ or uncertain between choices (LaClair and Lacreuse 2016; Tolman 1938), or were they comparing or learning the characteristics of stimuli (Gellermann 1933)? In the introduction, we argued that different patterns of variation of behavioural responses across blocks would be associated with these two possibilities at the within-phase level. Given that the squirrels decreased their response inhibition to the incorrect stimulus with increased training blocks (Fig. 3c, d), increased the number of head-switches across blocks in both discrimination and reversal phases (Fig. 3e, f) and increased head-switching rate is related to more correct choices than incorrect choices, our results appear to support the latter possibility.

**Fig. 4:**
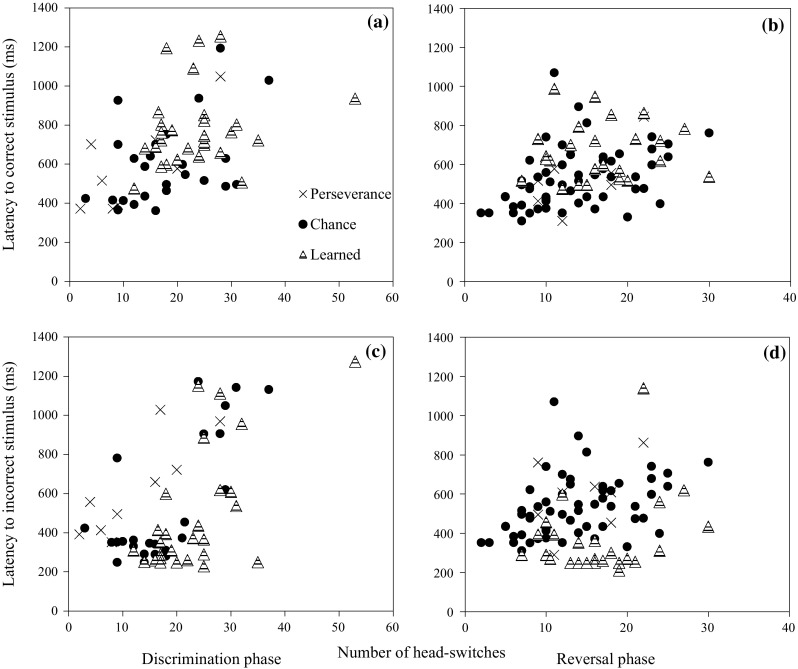
Relationships between behavioural responses. Response latency of first response to the correct stimulus (ms) and the number of head-switches per block in **a** the discrimination phase and **b** the reversal phase. Response latency of first response to the incorrect stimulus (ms) and the number of head-switches per block **c** in the discrimination phase and **d** the reversal phase

Despite this, it is still possible that squirrels were confused at the beginning of both training phases. When we examine the characteristics of behavioural responses in each learning stage for each training phase (Fig. 4a–d), the perseveration stages are associated with the highest response inhibition to the incorrect stimulus (Fig. 3c, d), comparable to the response inhibition to the correct stimulus (Fig. 3a, b), and also low head-switching (Fig. 3e, f). As argued in introduction, the fact that squirrels showed comparable response latencies to correct and incorrect stimuli, along with increased head-switching later in training suggests that they were confused at the beginning of each training phase. Once squirrels passed through this stage, errors in the chance-level stage showed lower response latencies to both stimulus than the previous stage. These results suggest that the squirrels learned through responding rapidly to a choice. Head-switching in this stage was more than the previous stage in the discrimination phase but less than the previous stage in the reversal phase (Fig. 3e–f), suggesting attention to the stimuli at this stage increased in the discrimination phase but lapsed in the reversal phase. In the learned stage, the response latency to the correct stimulus and the number of head-switches were highest, whereas incorrect response latencies were the lowest of all learning stages. These characteristics suggest that the remaining errors at this stage were due to failures of inhibition control or lapses in attention.

Although these behavioural responses may well reflect learning mechanisms such as inhibitory control and attention, it is not easy to disentangle attention from inhibitory control (e.g. Tait and Brown 2007) when seeking to account for poor learning performance or an increased number of error responses in a task. In our case, Squirrel 4 failed to reach the stringent learning criterion (75% for two consecutive blocks). When we tried to analyse why he could not do so, limited information was provided from his behavioural responses: neither his number of head-switches nor his response latencies towards the incorrect stimulus were significantly different from other squirrels that did reach the learning criterion. Across all the squirrels, the rate of learning in this experiment was slow, compared with the learning of spatial reversals studied by Chow et al. (2015). This may reflect a high level of spatial cognitive capacity in grey squirrels, due to their scatter-hoarding mode of life. Alternatively, the difference might be due to methodology. Although squirrels might show better performance in spatial ability than colour discrimination ability, the current study of colour reversal learning used a touch screen, whereas Chow et al. (2015) used a traditional object apparatus for the spatial reversal learning task. This difference may have masked their learning abilities in the colour reversal task. For example, O’Hara and colleagues (2015) showed that kea showed better learning performance on using solid object apparatus on ground than using images on touch screen. Given that squirrels excel in object manipulation, it would not be surprising if squirrels perform better in a solid object colour discrimination–reversal learning task. Indeed, Wills et al. (2009) examined three-dimensional colour and shape discrimination using solid objects and showed that squirrels could learn the task within 2 trials. A further possibility would be due to age: some studies have found that older individuals showed poorer performance in the reversal phase than younger individuals, to name a few, among rats (Brushfield et al. 2008), beagle dogs (Tapp et al. 2003) and rhesus monkeys (Bartus et al. 1979; Rapp 1990). However, in our case there was no obvious effect of age on learning performance: one younger subject, Squirrel 2 (aged 2), took a similar number of blocks as Squirrel 4 (aged 7) to reach the learning criterion in both training phases, and the oldest squirrel (Squirrels 1, aged 9) reached the learning criterion as fast as Squirrel 5 (aged 2) in both training phases (see Table S1 for squirrels’ background information). A final possibility is that the red/green discrimination was difficult for them: although they clearly could discriminate these colours, given that their colour vision is dichromatic, the difference between the colours would not have been large for them and hence may not have been salient.

In summary, we show that squirrels are capable of overcoming their pre-potent preferences and thus indicate behavioural flexibility. Our findings also provide further evidence that head-switching and the response latencies to correct and incorrect stimuli may be used as indices of attention and inhibitory control, respectively, for the simultaneous reversal learning task. Each behavioural response changed systematically across learning stages within the two training phases reflects that fewer errors (and hence more correct choices) require constant exhibition or increased inhibition control to the correct stimulus, along with increased exhibition of head-switching to enhance attention in comparing or learning the stimuli. In a broader context, both inhibitory control and attentional mechanisms likely have adaptive significance for grey squirrels, an exceptionally successful invasive species (Lowe et al. 2000) that are expected to show high flexibility in their behaviour, as has been shown in invasive birds (Sol et al. 2002). To what extent these behavioural responses are related to other ecologically relevant behaviours such as the response to conspecifics and heterospecifics during caching is largely unknown, although field studies reveal that squirrels stop digging and increase the latency to start caching when conspecifics are present (Hopewell and Leaver 2008), showing that they are attentive to the presence of conspecifics (Hopewell et al. 2008) and heterospecifics (Schmidt and Ostfeld 2008) during caching. Future studies could focus on the inter-correlation between these mechanisms in ecologically relevant contexts, so as to build a complete picture of the extent to which similar cognitive mechanisms support animals in adapting to change in natural environment.

## Electronic supplementary material

Below is the link to the electronic supplementary material.

Supplementary material 1 (DOCX 14 kb)

## References

[CR1] Alsiö J, Nilsson SRO, Gastambide F, Wang RAH, Dam SA, Mar AC, Tricklebank M, Robbins TW (2015). The role of 5-HT2C receptors in touchscreen visual reversal learning in the rat: a cross-site study. Psychopharmacology.

[CR2] Arnall S, Cheam LY, Smart C, Rengel A, Fitzgerald M, Thivierge JP, Rodger J (2010). Abnormal strategies during visual discrimination reversal learning in ephrin-A2-/- mice. Behav Brain Res.

[CR3] Bartus RT, Dean RL, Fleming DL (1979). Aging in the rhesus monkey: effects on visual discrimination learning and reversal learning. J Gerontol.

[CR4] Bates D, Maechler M, Bolker B, Walker S (2015). Fitting linear mixed-effects model using lme4. J Stat Softw.

[CR5] Bingman VP, Gasser B, Colombo M (2008). Responses of pigeon (*Columba livia*) Wulst neurons during acquisition and reversal of a visual discrimination task. Behav Neurosci.

[CR6] Birrell JM, Brown VJ (2000). Medial frontal cortex mediates perceptual attentional set shifting in the rat. J Neurosci.

[CR7] Boulougouris V, Glennon J, Robbins TW (2008). Dissociable effects of selective 5-HT2A and 5-HT2C receptor antagonists on serial spatial reversal learning in rats. Neuropsychopharmacology.

[CR8] Brady AM, Floresco SB (2015). Operant procedures for assessing behavioural flexibility in rats. J Vis Exp.

[CR9] Brushfield AM, Trinh Luu, Callahan B, Gilbert PE (2008). A comparison of discrimination and reversal learning for olfactory and visual stimuli in aged rats. Behav Neurosci.

[CR10] Brust V, Wuerz Y, Krüger O (2013). Behavioural flexibility and personality in Zebra Finches. Ethology.

[CR11] Bryce CA, Howland JG (2015). Stress facilitates late reversal learning using a touchscreen-based visual discrimination procedure in male Long Evans rats. Behav Brain Res.

[CR12] Bussey TJ, Muir JL, Everitt BJ, Robbins TW (1997). Triple dissociation of anterior cingulate, posterior cingulate, and medial frontal cortices on visual discrimination tasks using a touchscreen testing procedure for the rat. Behav Neurosci.

[CR13] Cardinal RN, Aitken MRF (2010). Whisker: a client-server high-performance multimedia research control system. Behav Res Methods.

[CR001] Carvalho LS, Cowing JA, Wilkie SE, Bowmaker JK, Hunt DM (2006) Shortwave visual sensitivity in tree and flying squirrels reflects changes in lifestyle. Curr Biol 16:R81–R83. doi:10.1016/j.cub.2006.01.045 10.1016/j.cub.2006.01.045 16461266

[CR14] Chow PKY, Leaver LA, Wang M, Lea SEG (2015). Serial reversal learning in gray squirrels: learning efficiency as a function of learning and change of tactics. J Exp Psychol Anim Learn Cogn.

[CR15] Chow PKY, Lea SEG, Leaver LA (2016). How practice makes perfect: the role of persistence, flexibility and learning in problem-solving efficiency. Anim Behav.

[CR16] Chudasama Y, Robbins TW (2003). Dissociable contributions of the orbitofrontal and infralimbic cortex to pavlovian autoshaping and discrimination reversal learning: further evidence for the functional heterogeneity of the rodent frontal cortex. J Neurosci.

[CR17] Clark L, Cools R, Robbins TW (2004). The neuropsychology of ventral prefrontal contex: decision-making and reversal learning. Brain Cogn.

[CR18] Clarke HF, Dalley JW, Crofts HS, Robbins TW, Roberts AC (2004). Cognitive inflexibility after prefrontal serotonin depletion. Science.

[CR19] Cole EF, Morand-Ferron J, Hinks AE (2012). Cognitive ability influences reproductive life history variation in the wild. Curr Biol.

[CR20] Colwill RM, Raymond MP, Ferreira L, Escudero H (2005). Visual discrimination learning in zebrafish (*Danio rerio*). Behav Processes.

[CR21] Dukas R (2013). Effects of learning on evolution: robustness, innovation and speciation. Anim Behav.

[CR22] Gellermann LW (1933). Form discrimination in chimpanzees and two-year-old children: i. Form (Triangularity) per se. Pedagog Sem J Genet Psychol.

[CR23] Griesbach GS, Hu D, Amsel A (1998). Effects of MK-801 on vicarious trial-and-error and reversal of olfactory discrimination learning in weanling rats. Behav Brain Res.

[CR24] Hopewell LJ, Leaver LA (2008). Evidence of social influences on cache-making by grey squirrels (*Sciurus carolinensis*). Ethology.

[CR25] Hopewell LJ, Leaver LA, Lea SEG (2008). Effects of competition and food availability on travel time in scatter-hoarding gray squirrels (*Sciurus carolinensis*). Behav Ecol.

[CR26] Hopewell LJ, Leaver LA, Lea SEG, Wills AJ (2010). Grey squirrels (*Sciurus carolinensis*) show a feature-negative effect specific to social learning. Anim Cogn.

[CR27] Hu D, Amsel A (1995). A simple test of the vicarious trial-and-error hypothesis of hippocampal function. Proc Natl Acad Sci.

[CR28] Hu D, Xu X, Gonzalez-Lima F (2006). Various trial-and-error behaviour and hippocampal cytochrome oxidase activity during Y-maze discrimination learning in the rat. Int J Neurosci.

[CR29] Isden J, Panayi C, Dingle C, Madden J (2013). Performance in cognitive and problem-solving tasks in male spotted bowerbirds does not correlate with mating success. Anim Behav.

[CR30] Izquierdo A, Jentsch JD (2012). Reversal learning as a measure of impulsive and compulsive behaviour in addictions. Psychopharmacology.

[CR31] Izquierdo A, Brigman JL, Radke AK, Rudebeck PH, Holmes A (2016). The neural basis of reversal learning: an updated perspective. Neuroscience.

[CR32] Jayne K (2014) Challenges faced by foraging Eastern grey squirrels, *Sciurus carolinensis:* competition, pilferage and predation risks. Retrieved from https://ore.exeter.ac.uk/repository/bitstream/handle/10871/15656/JayneK.pdf?sequence=1&isAllowed=y

[CR33] Jones B, Mishkin M (1972). Limbic lesions and problem of stimulus-reinforcement associations. Exp Neurol.

[CR34] Kelber A (1996). Colour learning in the hawkmoth *Macroglossum Stellatarum*. J Exp Biol.

[CR35] Kemble ED, Beckman GJ (1970). Vicarious trial and error following amygdaloid lesions in rats. Neuropsychologia.

[CR36] Knudson C (2015). glmm: generalized linear mixed models via Monte Carlo likelihood approximation. R package version.

[CR37] LaClair M, Lacreuse A (2016). Reversal learning in gonadectomized marmosets with and without hormone replacement: are males more sensitive to punishment?. Anim Cogn.

[CR38] Leal M, Powell BJ (2012). Behavioural flexibility and problem-solving in a tropical lizard. Biol Lett.

[CR39] Liedtke J, Schneider JM (2014). Association and reversal learning abilities in a jumper spider. Behav Processes.

[CR40] Lowe S, Browne M, Boudjelas S (2000) 100 of the world’s worst invasive alien species. Global Invasive Species Program. Available at http://www.gisp.org/publications/brochures/100worst.pdf

[CR41] Lucon-Xiccato T, Bisazza A (2014). Discrimination reversal learning reveals greater female behavioural flexibility in guppies. Biol Lett.

[CR42] Macdonald IMV (1997). Field experiments on duration and precision of grey and red squirrel spatial memory. Anim Behav.

[CR43] MacLean EL, Hare B, Nunn CL, Addessi E, Amici F, Anderson RC (2014). The evolution of self-control. Proc Natl Acad Sci.

[CR44] Muenzinger KF (1938). Vicarious trial and error at a point of choice: i. A general survey of its relation to learning efficiency. J Genet Psychol.

[CR45] Nicolakakis N, Sol D, Lefebvre L (2003). Behavioural flexibility predicts species richness in birds, but not extinction risk. Anim Behav.

[CR46] Nilsson SRO, Alsiö J, Somerville EM, Clifton PG (2015). The rat’s not for turning: dissociating the psychological components of cognitive inflexibility. Neurosci Biobehav Rev.

[CR47] O’Hara M, Huber L, Gajdon GK (2015). The advantage of objects over images in discrimination and reversal learning by kea, *Nestor notabilis*. Anim Behav.

[CR48] Raine NE, Chittka L (2012). No trade-off between learning speed and associative flexibility in bumblebees: a reversal learning test with multiple colonies. PLoS ONE.

[CR49] Rapp RP (1990). Visual discrimination and reversal learning in the aged monkey (*Macaca mulatta*). Behav Neurosci.

[CR50] Redish AD (2016). Vicarious trial and error. Nat Rev Neurosci.

[CR51] Schmidt KA, Ostfeld RS (2008). Eavesdropping squirrels reduce their future value of food under the perceived presence of cache robbers. Am Nat.

[CR52] Shettleworth SJ (2010). Cognition, evolution and behavior.

[CR53] Silver PH (1976). Grey squirrel spectral sensitivity by heterochromatic flicker and its implications. Vis Res.

[CR54] Sol D, Timmermans S, Lefebvre L (2002). Behavioural flexibility and invasion success in birds. Anim Behav.

[CR55] Sol D, Bacher S, Reader SM, Lefebvre L (2008). Brain size predicts the success of mammal species introduced into novel environments. Am Nat.

[CR56] Sol D, Lapiedra O, González-Lagos C (2013). Behavioural adjustments for a life in the city. Anim Behav.

[CR57] Tait DS, Brown VJ (2007). Difficulty overcoming learned non-reward during reversal learning in rats with ibotenic acid lesions of orbital prefrontal cortex. Ann NY Acad Sci.

[CR58] Tait DS, Brown VJ (2008). Lesions of the basal forebrain impair reversal learning but not shifting of attention set in rats. Behav Brain Res.

[CR59] Tapp PD, Siwak CT, Estrade J, Head E, Muggenburg BA, Cotman CW (2003). Size and reversal learning in the beagle dog as a measure of executive function and inhibitory control in aging. Learn Mem.

[CR60] Tolman EC (1938). The determiners of behavior at a choice point. Psychol Rev.

[CR61] Voytko ML, Olton DS, Richardson RT, Gorman LK, Tobin JR, Price DL (1994). Basal forebrain lesions in monkeys disrupt attention but not learning and memory. J Neurosci.

[CR62] Wills AJ, Lea SEG, Leaver LA (2009). A comparative analysis of the categorization of multidimensional stimuli: i. Unidimensional classification does not necessarily imply analytic processing; evidence from pigeons (*Columba livia*), squirrels (*Sciurus carolinensis*) and humans (*Homo sapiens*). J Comp Psychol.

[CR63] Winer BJ (1971). Statistical principles in experimental design.

