# Supplementary material for: Touch screen assays of behavioural flexibility and error characteristics in Eastern grey squirrels (Sciurus carolinensis)
Source: Anim Cogn. 2017 Jan 27;20(3):459–71. doi: 10.1007/s10071-017-1072-z (PMC5394141; doi:10.1007/s10071-017-1072-z)
Supplement: Supplementary file 1 — Supplementary material 1 (DOCX 14 kb) [file 10071_2017_1072_MOESM1_ESM.docx]

Supplementary materials for manuscript titled as ‘Touchscreen assays of behavioural flexibility and error characteristics in Eastern grey squirrels (*Sciurus carolinensis*).’ by

PKY Chow, L Leaver, M Wang and SEG Lea

Table S1. Information about the five squirrels. Age for each squirrel is estimated at the point of received, along with their status.

|  | Name | Sex | Estimated Age | Previous experience to use touch screen | Status |
| --- | --- | --- | --- | --- | --- |
| Squirrel 1 | Arnold | M | 9 | Y | Rescued |
| Squirrel 2 | Leonard | M | 2 | N | Hand-raised |
| Squirrel 3 | Sarah | F | 2 | N | Hand-raised |
| Squirrel 4 | Simon | M | 7 | Y | Rescued |
| Squirrel 5 | Suzy | F | 2 | N | Hand-raised |
